# Psycho-Physiological Stress Recovery in Outdoor Nature-Based Interventions: A Systematic Review of the Past Eight Years of Research

**DOI:** 10.3390/ijerph16101711

**Published:** 2019-05-16

**Authors:** Sus Sola Corazon, Ulrik Sidenius, Dorthe Varning Poulsen, Marie Christoffersen Gramkow, Ulrika Karlsson Stigsdotter

**Affiliations:** Department of Geosciences and Natural Resource Management, University of Copenhagen, Rolighedsvej 23, 1958 Frederiksberg C, Denmark; us@ign.ku.dk (U.S.); dvp@ign.ku.dk (D.V.P.); mg@ign.ku.dk (M.C.G.); uks@ign.ku.dk (U.K.S.)

**Keywords:** health-promoting environments, natural environments, mood, self-estimated stress, heart rate variability, cortisol, narrative synthesis, EPHPP quality assessment

## Abstract

Background: In modern, urban daily life, natural environments are increasingly recognized as an important resource for stress recovery and general well-being. Aim: the present review aims to provide an overview and synthesis of the past eight years’ research into the psycho-physiological effects of outdoor nature-based interventions, related to stress recovery. Method: a structured search was performed in seven databases, returning 5618 articles. Removal of duplicates and initial screening gave a total of 95 studies. After full text reading, 36 studies were included in the assessment. Results: most of the psychological outcomes were related to different emotional measures. The synthesis of the results points towards outdoor, nature-based exposure having a positive effect on different emotional parameters, related to stress relief. The studies into physiological measures showed more equivocal results. Conclusion: the research, conducted over the past eight years, into outdoor, nature-based exposure has now attained a sound evidence base for psychological and especially emotional effects, but the evidence base for physiological effects within this timeframe shows a great degree of heterogeneity. Limitations: interpretation of the results is limited by the review only covering the past eight years’ research on the subject.

## 1. Introduction

According to the World Health Organization, stress has become a serious global health risk in modern, urban daily life [1]. Since over half the world’s population now lives in urban areas, it is vital to find ways of promoting stress recovery in daily life [2]. One method that is receiving increased attention is spending time in nature. Natural environments are today acknowledged as an important public health resource for promoting stress recovery and general well-being [3,4].

The research has mainly come from European and North American research institutions. However, more and more studies are being carried out in Asian countries, especially Japan and South Korea, where the concept of shinrin-yoku (‘forest bathing’), first proposed in 1982, is becoming increasingly popular and scientifically recognized [5].

Several recent reviews have been conducted on the health benefits of nature, focusing on various health-related parameters and environments [6,7,8,9,10]. A review by McMahan and Estes [6] included measures of negative and positive affect in a broad range of natural environments, while Haluza, Schönberger, and Cervinka focused physiological effects [7]. A review by Kondo, et al. [8] included all outdoor environments, not just natural environments, while James, et al. alone focused on neighborhood greenness [9]. A review by Twohig–Bennet and Jones (2018) [10] included both observational and interventional studies as well as a broad range of health-related measures. The reviews adjoin and overlap with one another even though each is unique in scope, contributing to an increasingly comprehensive understanding of the possible health effects of human–nature interactions. Overall, they find positive associations between natural environments and various aspects of human health. However, they also reach similar conclusions to the effect that the evidence base remains limited by study designs and/or high levels of heterogeneity.

The present review seeks to provide an overview of the past eight years’ research into psycho-physiological effects, related to stress recovery, of outdoor green nature exposure. The rationale for the limited time frame was mainly directed by limited resources (funding), and the specific timeframe was chosen because the last review of the psychological and physiological effects of nature exposure was published by Bowler et al. [11] in 2010. However, it should be highlighted that the present review is not a direct continuation of the review by Bowler et al., which compared the effects of natural environments with those of synthetic environments and had a boarder scope concerning health. 

### 1.1. Psychological and Physiological Stress Recovery

Stress arousal is human beings’ natural response to a strain that is appraised as potentially threatening and that gives rise to negative emotions [12]. The appraised threat starts a cascade of physiological responses to mobilize energy: steroid hormones are released in the endocrine system, and the sympathetic nervous system is activated, which affects cardiovascular functioning and increases heart rate and perspiration [13].

According to Roger Ulrich’s environmental and psycho-psychological stress recovery theory [14], stress recovery involves both physiological and physiological components. Physiological recovery entails a shift to parasympathetic nervous activity, which sustains the organism’s healthy functions in the cardiovascular, endocrine, and immune system, whereas psychological recovery entails a positive change in emotional state.

Recovery of cognitive functioning, as set forth in Steven and Rachel’s Attention Restoration Theory [15], can also be seen as related to stress recovery. However, as the latest reviews on the cognitive effects of natural environments have been published quite recently [16,17], it was decided not to include this dimension in the present review and to instead focus on psychological and physiological aspects of stress recovery.

### 1.2. Research Questions

What is the latest evidence base for psychological effects, related to stress recovery, by nature exposure?

What is the latest evidence base for physiological effects, related to stress recovery, by nature exposure?

## 2. Method

The methodology of the systematic review followed the guidelines set forth in the Preferred Reporting Items for Systematic Review and Meta-Analysis Protocols (PRISMA-P) [18]. The PICO (Population/Problem, Intervention, Comparison, Outcome) framework (Table 1) was used to clarify the objectives of the review and facilitate the search strategy. (The full protocol in Danish can be acquired by contacting the corresponding author.)

### 2.1. Eligibility Criteria

We decided to include both randomized controlled trials (RCT) and non-randomized controlled trials. The rationale behind this decision was that the field largely still consists of non-randomized trials, and excluding non-RCT studies might thus result in an incomplete summary of the status. This is a common approach in reviews, although one should be aware of the greater risk of bias in the non-RCT studies [19] due to the less rigorous study design.

The language was limited to English and the research to peer-reviewed studies published between January 2010 and March 2018. These limitations were mainly directed by limited resources (funding, time, and translators). Limitations concerning language and peer-reviewed studies are common in reviews, but they subject the results to limitations and potential biases [20], which will be discussed in the limitations section.

To be eligible, the studies should have adult informants without mental disabilities or serious physical or mental illnesses not related to stress. The exposure should take place outdoors in natural green environments, whether in urban gardens and parks or in more remote and unspoiled areas such as forests, mountains, grasslands, and beaches. It was decided to include only outdoors nature-based interventions to obtain some consistency across studies on the environmental variable. Further studies comparing, for example, real nature and lab stimulation thereof have obtained heterogeneous results [21,22], which calls into question the transferability of results between the two settings. The same rationale of consistency in the environmental variable also led to the exclusion of blue environments such as the sea, lakes, and rivers. Only studies with sedentary and light physical activity were included, as vigorous physical exercise, such as running or mountain biking, have been found to have psychological and physiological effects in themselves [23].

To be eligible, the studies needed to entail psychological effect measures in terms of measuring changes in emotional states and/or physiological effect measures in terms of measuring changes in cardiovascular, endocrine, and/or immune functioning, in accordance with the stress recovery theory (SRT) [14]. Studies only using outcome measures, not validated through research, concerning their psychometric properties [24], were excluded. If a study entailed both validated and non-validated outcome measures, it was included, and only the validated measures were reported in the review.

### 2.2. Information Source and Search Strategy

A structured search was performed in the following seven databases: PUBMED, Web of Science, PsycInfo, SCOPUS, ASSIA, CINAHL, and Cocraine. The search took place in April 2018. The search string used OR to search for different physiological and psychological outcomes and different nature-based interventions respectively.

Outcome measures of emotional states, related to stress recovery, were operationalized into the following search terms: stress OR recovery OR health OR restorat OR well-being OR {well being} OR wellbeing OR well-being OR burnout OR fatigue OR emotion* OR affect* OR feeling* OR mood OR relax*. Outcome measures of cardiovascular, endocrine, and/or immune functioning were operationalized into the following search terms: cardiovascular OR {blood pressure} OR heartrate OR {heart rate} OR endocrine OR immune OR physiological* OR cortisol OR noradrenaline OR adrenaline OR dopamine.

The possible outcomes were combined with possible outdoors nature-based interventions in the search string by AND.

Search terms for outdoors green nature-based interventions were natur* OR green OR outdoor OR forest OR wilderness OR wood* OR garden OR park OR horticultur* OR {open space*} OR vegetation* OR seaside OR {sea side}.

When possible, depending on the individual search engine, the subject was limited to human and the areas to psychology, social sciences, nursing, arts and humanities, medicine, multidisciplinary research, and health professions.

### 2.3. Inclusion and Assessment

The identified articles were screened for eligibility by title and, in case of doubt, by reading the abstract. Assessment of the included studies was based on the quality assessment tool for quantitative studies developed by the Effective Public Health Practice Project (EPHPP) [25]. This instrument provides an overall methodological rating of studies as strong, moderate, or weak based on eight parameters: selection bias, study design, confounders, blinding, data collection methods, withdrawals and dropouts, intervention integrity, and analysis. The parameters are individually rated as strong, moderate, or weak. If a study has two or more weak ratings in the parameters, it is considered weak overall. Studies with only one weak rating are considered moderate, and only studies with no weak ratings are considered strong. The tool has been evaluated for construct validity and inter-rater reliability [26]. Each study was assessed independently by two researchers using the EPHPP tool. The individual assessments were discussed between the research team (authors), and a final rating was granted to each study based on agreement between researchers. In order to handle possible duplicates, it was decided that if the same study was reported in several papers, the papers would be included if they involved different effect measures related to the scope of the review. In case of duplication of results, only the results from the first-published paper would be included. 

## 3. Results

### 3.1. Article Selection Process

**Figure 1:**
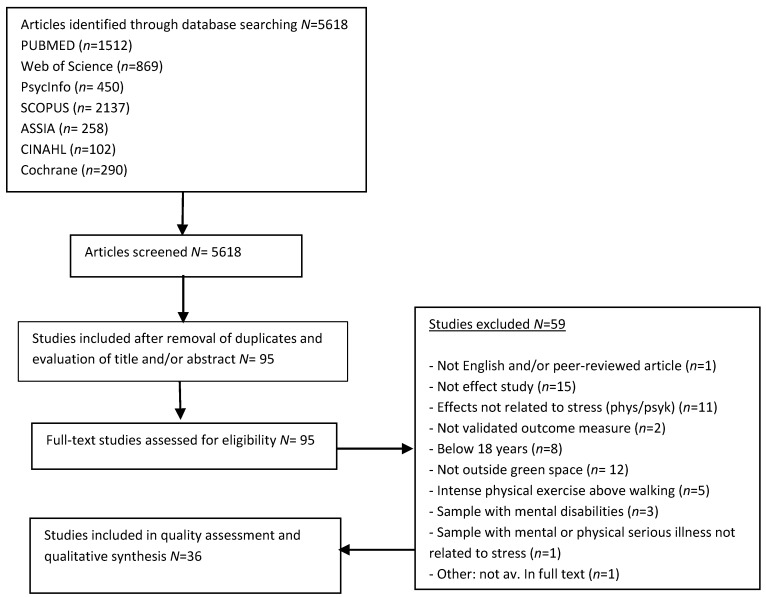
Flow diagram of article-selection process.

The database search returned 5618 articles. Removal of duplicates and initial screening gave a total of 95 studies. After full-text reading, 59 studies were excluded, and 36 studies were included in the assessment and synthesis (Figure 1).

### 3.2. Characteristics of Included Studies

Table 2 summarizes the characteristics of the included studies: country of origin, study design, sample size and characteristics, type of intervention and control, time duration, and quality assessment.

#### 3.2.1. Study Design and Quality Assessment

Three studies were conducted as randomized controlled trials, with one being a crossover RCT. In the quality assessment, two of the studies were assessed as moderate and one was assessed as weak. Seventeen of the studies were categorized as controlled clinical trials (CCT), and nine of these had crossover designs. Five of the studies were assessed as weak, and three were assessed as moderate in quality. Sixteen studies had pre-post designs, with five including a control group and three in the form of a crossover design (Table 2). Only one of the pre-post studies was assessed as moderate in the quality assessment; the remaining fifteen studies were assessed as weak.

None of the included studies were considered strong overall according to the EPHPP quality assessment (Table 2, last column). This was due to several recurring weak ratings in the parameters: lack of information on recruitment procedure and/or using self-referred individuals as a sample (selection bias), lack of information on withdrawals and dropouts (withdrawals and dropouts), and not including comprehensive background information on the subjects (confounders). The issue of potential confounders in the EPHPP relates to relevant confounders between groups. In the studies with no control group or a crossover design, we modified the questions to concern the level of background information on the single group. Furthermore, none of the studies provided information on blinding of either assessors or informants. The parameter concerning blinding of informants to the research question might not be applicable in studies with multiple environmental exposures. However, the parameter is relevant to the blinding of the assessors. To allow for an alternative assessment of the blinding parameter issues, we carried out a second overall quality assessment excluding this parameter (Table 2, last column).

#### 3.2.2. Location 

Most of the studies were conducted in Europe (14) and Asia (14), followed by the USA (6), Canada (1), and Australia (1). A variety of European countries were represented by one study each (Denmark, Iceland, Finland, Ireland, Germany, the Netherlands, Austria, and Lithuania), two studies were from Sweden, and four studies were from the UK. In Asia, Japan was represented with nine studies, making it by far the single most-represented country in the review. South Korea, China, and Taiwan were each represented with one study (Table 2). 

#### 3.2.3. Sample Characteristics

The sample sizes varied widely, ranging from 9 to 935 subjects in the RCTs, 9 to 418 subjects in the CCTs, and 10 to 50 subjects in the pre-post studies (Table 2). Great variation was also found in sample populations, which included office workers; allotment gardeners; veterans; elderly subjects; and subjects with specific mental and physical illnesses related to stress, depression, and cardiovascular disease. The most common sample consisted of university students (10). Most studies included both sexes as subjects (22).

#### 3.2.4. Environments and Activities

The most common research setup entailed comparing an intervention in a natural environment, forest, or park to an intervention in an urban environment, mainly in city centers (12). The most common activity was walking and/or sitting (22). A few studies involved other activities such as gardening [30,37,54] and relaxation exercises in nature [53,54,59,60]. The durations of the interventions differed substantially and were spread between 15 and 55 min (14), one to several hours (3), one to several days (8), one to several weeks (3), and months (8) (Table 2).

### 3.3. Summary and Synthesis of Psychological Outcomes

The heterogeneous data, wide range of interventions and study designs, generally low quality of studies (low: 31, moderate: 5, strong: 0), and recurring missing information in the results sections made the studies unsuitable for pooling for meta-analysis [63]. The findings were therefore first summarized and then compared in a narrative synthesis [64]. This was done separately for the psychological and physiological measures. An overview of the physiological measures and findings is presented in Table 3.

#### 3.3.1. Stress, Burnout, and Recovery Outcomes

Eight studies included measures of the level of self-perceived stress (Table 3). Four studies found a significant difference between the intervention and control, in favor of the natural environment (nature ˃ control) [31,33,42,51], with significance levels between *p* ˂ 0.05–0.001. Three of the studies were CCTs. Two pre-post studies without control groups found no significant decrease from before to after the intervention [53,54]. There was an effect of condition (nature ˃ control) in the CCT study that included a stress recovery measure [46].

#### 3.3.2. Emotional Outcomes

In the studies including measures of positive and negative affect (Table 4), eight studies—four of them CCTs—found a positive significant difference in the pre-post measures of the intervention [36,37,38,46,47,51,53,56], and two studies—one RCT and one pre-post without control group—found no significant difference in the pre-post measures of the intervention [27,55].

Seven studies—four CCTs and three pre-post—found positive significant differences in pre-post measures of total mood disturbance or on subscales [29,32,34,45,48,58,59]. Two CCT studies found positive significant differences in both the intervention and control [34,41]. Two studies—one CCT and one pre-post—found no significant difference in the pre-post measures [39,54]. Of the three studies using anxiety measures, three found a significant decrease from the intervention [29,61,62] and one found a significant difference between intervention and control [44].

The five studies including measures of depression [50,51,55,61] were all pre-post studies. Three of the studies reported a significant decrease from the intervention (nature ˃ control) [50,51,61], with significance levels of *p* < 0.001–0.001. One pre-post study with a combined depression, anxiety, and stress scale also found a significant decrease from the intervention [52].

### 3.4. Well-Being, Quality of Life, and Mental Health Outcomes

Five studies included measures related to well-being, quality of life, and mental health (Table 4). Four reported significant increases from the intervention [54,56,57,61], with significance levels of *p* ˂ 0.001–0.000, and one study found no significant effect [54].

#### 3.4.1. Synthesis of Psychological Outcomes

**Figure 2:**
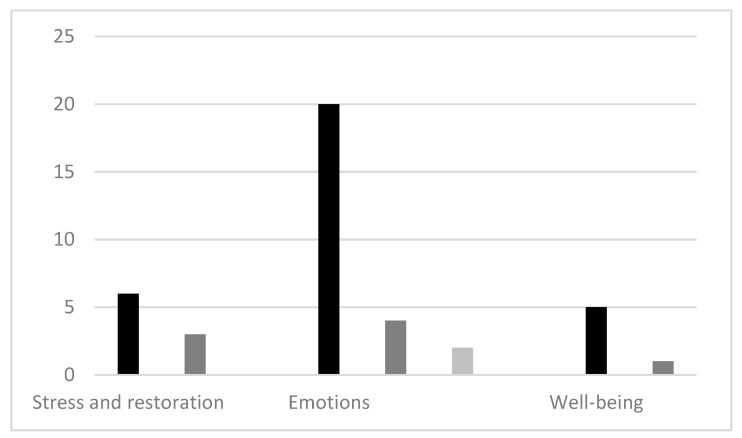
Synthesis of psychological outcomes of nature-based interventions. 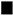 Number of studies with positive significant difference in pre-post measures of the nature intervention alone and/or effect of condition favoring the nature intervention. 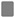 Number of studies with no significant difference found in pre-post measures of the nature intervention. 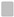 Number of studies with positive significant difference in pre-post measures of both intervention and control and/or no significant difference between intervention and control. Note: some of the studies have several different measurement instruments and are thus represented in multiple columns in the figure.

The most studied psychological outcomes were related to different measures of emotional change (Figure 2). Eighteen studies [29,32,34,36,37,38,44,48,50,51,53,56,58,61,62], including one RCT and eight CCTs, found positive significant difference on different measures of emotional change (significance levels *p* < 0.05–0.001), whereas only four studies [27,39,54,55] found no significant differences in the pre-post measures. This points towards a coherent and largely unambiguous evidence base of the past eight years of nature-based interventions as having a positive effect on various emotional parameters related to stress recovery. The evidence base concerning perceived stress level measures is weaker, though mainly positive. Four of the five studies that found a significant decrease had a CCT design [31,33,42,46], which has greater weight than the three pre-post studies without control groups that did not find significant changes [53,54,61]. The lack in evidence base is therefore mostly due to the low number of studies and generally low significance levels (31,33,42: *p* < 0.01–0.05). The effects on well-being and quality of life had high significance levels in the five measures showing positive significant increase (*p* < 0.001–0.000) [54,56,57,61], and two of the studies also had large sample sizes (57: sample size 195; 56: sample size 935). However, these lacked control groups, which weakens the results. The evidence base for this aspect can therefore be regarded as promising, though lacking studies with control groups.

### 3.5. Summary and Synthesis of Physiological Outcomes

The findings are first summarized for the individual measures and then compared in a narrative synthesis [63]. An overview of the physiological measures and findings is presented in Table 4.

#### 3.5.1. Endocrine Outcomes

Ten studies, including one RCT and six CCTs, reported a significant decrease in cortisol levels and other stress hormones after the intervention [28,30,37,39,41,43,46,48,58,59] (Table 4). They all had low significance levels: *p* ˂ 0.05–0.01. Three of the CCT studies also found a significant decrease in the pre-post measures of the control exposure [37,39,46]. Four studies found no significant difference in the pre-post measures of the intervention [27,32,34,49]. The studies that compared the effect of the intervention to that of the control also showed divergent results: three of the studies, all CCTs, found no significant difference [31,41,45], whereas three studies, two of which were CCTs, found a significant difference in favor of the intervention [40,44,50].

The use of cortisol as a measurement instrument was applied inappropriately in two of the studies [28,30], as it only was measured twice (pre-post) in interventions spanning 10 weeks [30] and three months [28], respectively. Pre-post cortisol measures are only appropriate for assessing acute responses to stress in short interventions or in interventions with long time spans to establish a pattern in cortisol levels by repeated measures several times a day over a number of days to achieve a reliable estimate [64].

#### 3.5.2. Cardiovascular Outcomes

Nine studies used heartrate variability as a measure of cardiovascular change (Table 4). One CCT study found a significant change in the pre-post measures of the intervention [43], and three studies—one CCT and two pre-post with control groups—found a significant difference between groups in favor of the intervention [40,44,50]. Two CCT studies found positive significant differences of both intervention and control as well as no difference between groups [41,45]. Two studies found no significant differences [39,62]. An inappropriate use of the HRV (heart rate variability) measure was detected in one study [50], in which the HRV post measures were collected a week after the intervention ended (six-week intervention with lunch break walk, with non-stressed university students as subjects).

Seven studies, including one RCT, found a significant decrease in intervention pre-post measures of blood pressure [27,41,45,49,58,60,62]. However, two of these studies—CCTs—also found a significant decrease in the control group and no significant difference between groups [41,45], while three of the studies lacked a control group [58,60,62]. Two studies found no significant decrease in the pre-post measures of the intervention [34,48]. The significance levels were low in all the studies (*p* ˂ 0.05–0.01).

The six studies that included pulse rate measures also showed divergent results: two studies without control groups found a significant decrease [58,62], whereas one study found no significant effect [60]. Two CCT studies found a difference between groups in favor of the natural environment [34,48], and one CCT study found no difference between groups [31]. The significance level for the effect was low in all the studies (*p* ˂ 0.01).

#### 3.5.3. Immune Outcomes

Two CCT studies included measures of immune functioning [32,42]. They found a difference in all measures in favor of the natural environment. None of the studies reported the effect of the intervention itself, and one study did not obtain baseline measures [42].

#### 3.5.4. Synthesis of Physiological Outcomes

**Figure 3:**
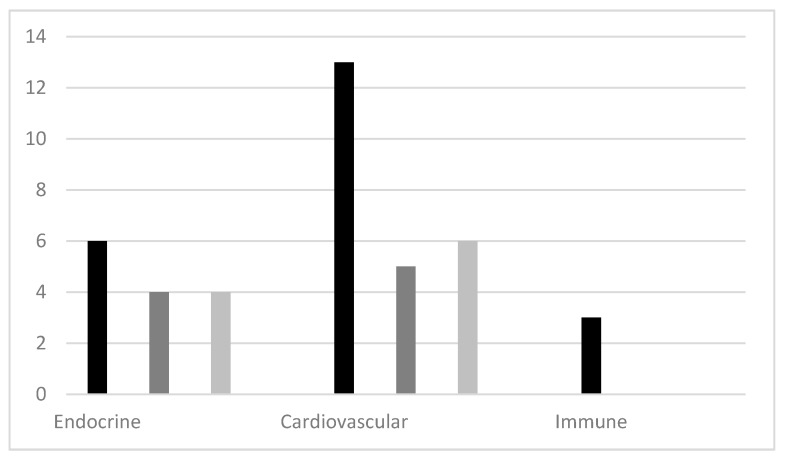
Synthesis of physiological outcomes of nature-based interventions. 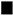 Number of studies with positive significant difference in pre-post measures of the nature intervention alone and/or effect of condition favoring the nature intervention. 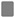 Number of studies with no significant difference found in pre-post measures of the nature intervention. 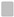 Number of studies with positive significant difference in pre-post measures of both intervention and control and/or no significant difference between intervention and control. Note: Some of the studies have several different measurement instruments and are thus represented in multiple columns in the figure.

The studies of endocrine and cardiovascular measures show highly heterogeneous results: the numbers of studies showing a significant decrease in pre-post measures and studies reporting no significant difference in pre-post measures and/or no significant differences between intervention and control groups were almost equal (Figure 3). Studies without control groups [58,59,60,62] were heavily represented in the measures showing significant decreases. As several of the CCT studies found significant decreases in pre-post measures of both the intervention and control and/or no significant difference between them [31,35,37,39,41,46], the results of the pre-post studies without control groups must be questioned in terms of whether or not the positive effect was caused by exposure to the natural environment. The positive results on immune functions stem from only two studies [32,42], one of which lacked baseline measures [42]. It is therefore impossible to draw any conclusions based on this outcome measure.

## 4. Discussion

### 4.1. Quality of the Studies

The included studies were comprised of study designs using self-referred individuals (24), many of whom were university students (10), as well as pre-post designs without randomization (15). Only three of the included studies were categorized as randomized controlled trials [27,28,29]. Information on several methodological aspects was generally missing in the studies. The past eight years’ research into the subject must therefore be considered quite methodologically weak overall. This conclusion on the general weakness in methodology is similar to that reached in other recent reviews with slightly different scopes [6,7,8,9,10,11], namely, that more studies using rigorous and transparent methodologies and study designs are needed. Only two studies reported effect sizes. This can be seen as a fundamental weakness in both the included studies and the review, relying solely on *p* values to evaluate the findings. As *p* values can only give information on the statistical significance of the found effect, related to the null hypotheses, and not the importance (how strong the effect is), they cannot be used to determine the therapeutic relevance of the given intervention [65]. However, one should also be aware of the potential biases when using effect sizes to perform meta-analyses on the strength of the effect across studies. It is therefore not recommended in reviews with studies assessed as weak and/or with clinical and methodological heterogeneity [63], which is the case for the present review as well as for most previous reviews.

### 4.2. Findings and Evidence Base

Based on the homogeneous, substantial, and statistically significant findings concerning emotional change, the evidence base for outdoor natural environments promoting this aspect of stress recovery seems sound. However, the limitation of being unable to determine the size of the effect and related therapeutic relevance as well as the assessment of the studies as generally weak should be taken into account. The evidence concerning the effects of exposure to nature on lowering self-perceived levels of stress is largely positive yet weak in the sense that there is a low number of studies and that the findings have low significance levels. The same applies to the evidence on various aspects of well-being and quality of life, which showed positive results but involved few studies, which themselves lacked control groups even though they had large sample sizes and high significance levels. Only one study measured recovery directly [46]. It showed a significant decrease and reported a large effect size. This could thus be an interesting measure, worthy of future research, especially given that the field seems saturated with measures of emotional change.

The evidence of the physiological effects related to stress recovery of the included past eight years’ nature-based interventions is more equivocal. The synthesis showed very heterogeneous results with regard to the effect on both endocrine and cardiovascular measures and very few measures of immune functioning. Due to the findings in studies with control groups (showing effect for both the intervention and control and/or no difference), it is recommended not to conduct studies with physiological measures without control groups. There are many possible explanations as to why positive outcomes were also found in control groups. One could be that the control condition was also effective or that the measurement instruments were insufficiently sensitive to detect differences. Of course, it could also simply be related to measurement errors or inappropriate use of the measurements, as was seen in two studies using cortisol measures only twice in interventions with long time duration [28,30] and one study using HRV as a post measure, a week after the intervention stopped [50].

The question remains why the physiological measures showed highly heterogeneous results. One explanation could be that the measures were insufficiently sensitive to capture the physiological effect of environmental exposure. Maybe there is a need to induce a stressor to detect significant physiological differences: a review of attention recovery found greater effect sizes when participants were induced with cognitively demanding tasks prior to exposure [17]. Another possibility is to raise the scientific level in the use of the physiological measures to achieve valid results. For example, take cortisol measures several times a day over the course of several days in interventions over a longer time span [64].

In addition, the relationship between psychological and physiological measures requires further study as the results are ambiguous. Studies using both measures find divergent results on psychological and physiological effects related to stress recovery.

### 4.3. EPHPP as Quality Assessment Tool

In the present review, the EPHPP tool was chosen as it is a validated and widely used assessment tool in health research [26] and has been used in previous reviews in the field [11,16]. In the EPHPP, the assessment of quality is based on eight parameters. However, it also includes questions on units of allocation and units of analysis as well as intervention integrity, which concerns whether there is a risk of having received an unintended intervention. These questions are not part of the quality rating. However, these aspects could cause potential bias as all studies used the individual as a unit of analysis, even though a substantial number had groups as the unit of allocation. Furthermore, the studies that had long time spans with short, repeated nature exposures had a high risk of the informants receiving an unintended intervention by factors outside the nature exposure. It is therefore recommended to use more comprehensive assessment tools to assess risk of bias in future studies, for example, the Cochrane tool for RCT studies [66] and the ROBINS-I tool for non-randomized studies [67].

### 4.4. “Nature-Positive” Bias

The field is dominated by the use of self-referred subjects, who might be expected to have an interest in nature and natural environments. This could therefore have caused a potential “nature-positive” bias. Haga, Halin, Holmgreen, and Sörqvist [68] have scientifically demonstrated the “nature-positive” bias in psychological recovery in a study in which participants heard the same soundtrack while performing a cognitively demanding task. One group was told it was the sound of a waterfall, while the other was told that it was from an industrial building site. The findings showed that the “waterfall group” reported significantly more recovery than did the “building site group”. As the setup in most studies in the review is built-up environments versus nature using self-referred individuals, there is strong cause for suspecting that the results are subject to “nature-positive” bias.

### 4.5. Limitations

This review has several limitations, one concerns only including published peer-reviewed research, which one could assume favors studies with significant findings and positive effects (publication bias) [69]. The large heterogeneity and non-significant results in the reported outcomes of the physiological measures in particular, however, do not support the idea that only nature-positive studies are prone to be published. However, this is a possible limitation of the study that one must take into account. On the other hand, including non peer-reviewed studies raises other potential biases, as the peer review process can be seen as a scrutiny filter for the scientific quality [70].

Another limitation concerns the inclusion of both randomized and non-randomized studies. This is a common approach in reviews, but it presents challenges when comparing studies, and it raises the risk of bias in the non-randomized studies [19]. In addition, the limitation concerning only English language studies being included raises a limitation by possibly missing out on important studies published in other languages [20].

The greatest limitation concerns the timeframe of the review. It should be seen as a limited perspective, only covering the past eight years’ research, and it raises the potential bias of studies with important contributions being published before this timeframe.

## 5. Conclusions

The past eight years’ research into psycho-physiological stress recovery in outdoors nature exposure supports the evidence of psychological effects, especially concerning emotional change. More ambiguous results were found regarding physiological effects. Therefore, the evidence base for the physiological effects of nature exposure in relation to the past eight years’ research must be regarded as quite weak. The general use of self-referred individuals imposes a potential strong bias. The results of the review should be seen in relation to its limitation concerning the relatively short timeframe.

## Figures and Tables

**Table 1:** Population, intervention, comparison, and outcome variables.

P	I	C	O
Population/Problem	Intervention	Comparison	Outcome
Adults (18+), all ethnicities, all countries, with or without stress-related issues/illness, without mental disabilities or other serious physical or mental illness.	Exposure to all types of outdoors natural green environments, with all types of sedentary and light exercise activities, in all time durations. From 2010–2018.	All kinds of comparisons or no comparison group.	Physiological (cardiovascular, endocrine, and immune) and psychological (mood, physiological stress, and well-being) outcomes related to stress prevention or stress treatment.

**Table 2:** Characteristics of included studies.

Main Author, Year	Country	Study Design	Sample Size	Characteristics	Age	Gender	Intervention	Control	Duration	Quality
			(Control)							Assessment *
[27] Grazuleviciene, 2016	Lithuania	RCT	10 (10)	Coronary artery disease	45–75 y	Mixed	Park walks	Urban walks	7 days	Weak (II)
[28] Razani, 2018	USA	RCT 2:1 ratio	50 (28)	Low-income parents		Mixed	Facilitated park tours	No facilitated tours	3 months	Moderate (III)
[29] Niedermeyer, 2017	Austria	RCT crossover	42 (42)	Healthy individuals	M:32 y	Mixed	Mountain hiking	Treadmill/sitting	3 h	Weak (II)
[30] Han, 2016	South Korea	CCT	14 (14)	Elderly with poor mental health	NA	NA	Horticultural therapy	Passive control	10 weeks	Weak (II)
[31] Kjellgren, 2010	Sweden	CCT	9 (9)	Individuals suffering from stress	37 y	Mixed	Relaxation in nature	Slideshow of nature	30 min	Moderate (II)
[32] Mao, 2011	China	CCT	10 (10)	University students	NA	Male	Stay/walk in forest	Stay walk in city	2 days	Weak (I)
[33] Largo-Wight, 2017	USA	CCT	18 (19)	University office staff	M:49 y	Mixed	Daily outdoor break	Daily indoor break	4 weeks	Moderate (III)
[34] Lee, 2010	Japan	CCT	12 (12)	University students	M:21 y	Male	Sitting in forest	Sitting in urban park	15 min	Weak (I)
[35] Olafdottir, 2017	Iceland	CCT	20 (24)	Inactive university students	NA	NA	Nature walk	Treadmill/nature videos	40 min	Weak (I)
[36] Passmore, 2014	Canada	CCT	43 (41)	Undergraduate students	NA	Mixed	Own choice nature activity	Own choice activity	2 weeks	Weak (I)
[37] Van den Berg, 2011	Netherlands	CCT	14 (16)	Allotment gardeners	38–79 y	Mixed	Gardening	Indoor reading	30 min	Weak (II)
[38] Fuegen, 2018	USA	CCT crossover	181 (181)	University students	M:22 y	Mixed	Green walk/green rest	Treadmill/rest	15 min	Weak (I)
[39] Gidlow, 2015	UK	CCT crossover	38 (38)	Unstressed adults	M:41 y	Mixed	Green walk/blue walk	Urban walk	30 min	Weak (II)
[40] Gladwell, 2016	UK	CCT crossover	13 (13)	Healthy individuals	M:39 y	Mixed	Nature walk	Campus walk	30 min	Weak (II)
[41] Horiuchi, 2014	Japan	CCT crossover	15 (15)	Healthy volunteers	M:36 y	Mixed	Sit and view in forest	View a curtain	15 min	Weak (I)
[42] Im, 2016	South Korea	CCT crossover	41 (41)	Undergraduate students	18–35 y	Mixed	Forest exposure	Urban exposure	2 h	Weak (I)
[43] Kobayashi, 2017	Japan	CCT crossover	408 (408)	Young individuals	NA	Male	Forest viewing	Urban viewing	15 min	Weak (I)
[44] Song, 2015	Japan	CCT crossover	23 (23)	University students	M:22 y	Male	Park walk	City walk	15 min	Weak (I)
[45] Stigsdotter, 2017	Denmark	CCT crossover	51 (51)	University students	20–36 y	Female	View and walk in arboretum	View and walk in city	55 min	Weak (II)
[46] Tyrväinen, 2014	Finland	CCT crossover	77 (77)	Workers in Helsinki	30–61 y	Mixed	View and walk in woodland	View and walk in city	45 min	Moderate (III)
[47] Berman, 2012	USA	pre/post crossover	20 (20)	Depressive disorder	M:26 y	Mixed	Park walk	Urban walk	50 min	Weak (I)
[48] Li, 2016	Japan	pre/post crossover	19 (19)	Middle-aged	40–69 y	Male	Guided walks in forest park	Urban guided walks	1 day	Weak (I)
[49] Toda, 2013	Japan	pre/post crossover	20 (20)	Volunteers	64–74 y	Male	Woodland walk	Sitting in office	45 min	Weak (I)
[50] Bang, 2017	South Korea	pre/post crossover	51 (48)	University students	M:26 y	Mixed	Forest walk in lunch break	Passive control	6 weeks	Weak (II)
[51] Marselle, 2014	UK	pre/post 2 groups	15 (16)	Adults	NA	NA	Group walks in nature	Passive control	13 weeks	Moderate (III)
[52] Bird, 2015	Australia	pre/post	20	Veterans	31–61 y	Male	Outdoor therapy program		6 days	Weak (I)
[53] Duvall, 2014	USA	pre/post	98	Veterans	20–49 y	Mixed	Nature-based recreation		4–7 days	Weak (II)
[54] Hofmann, 2017	Germany	pre/post	85	Volunteer gardeners	25–70 y	Mixed	Urban gardening		6 months	Weak (II)
[55] Iwata, 2016	Ireland	pre/post	15	Mental ill health	32–72 y	Mixed	Forest walk		13 weeks	Weak (I)
[56] Marselle, 2016	UK	pre/post	935	Elderly	55–74 y	Mixed	Walk in nature		13 weeks	Weak (I)
[57] McCaffrey, 2016	USA	pre/post	195	Adults with stress	N/A	Mixed	Garden walks		6 weeks	Weak (II)
[58] Ochiai, 2015a	Japan	pre/post	9	Normal-to-high blood pressure	40–72 y	Male	Relax and walk in forest		1 day	Weak (I)
[59] Ochiai, 2015b	Japan	pre/post	17	Middle aged	40–73 y	Female	Forest therapy program		2 days	Weak (I)
[60] Ohe, 2017	Japan	pre/post	43	Office workers	20–70 y	Mixed	Forest therapy program		2 days	Weak (I)
[61] Sahlin, 2015	Sweden	pre/post	57	On sick leave due to stress	45 y	Mixed	Nature-based therapy		16 weeks	Moderate (III)
[62] Yu, 2017	Taiwan	pre/post	128	Middle-aged and elderly	45–86 y	Mixed	Forest bathing program		2 h	Weak (I)

* The alternative quality assessment, which excludes the blinding parameter (Table 4), is included in the Quality Assessment column with the symbols I: low; II: medium; III: strong. Abbreviations. RCT: randomised controlled trial; CCT: controlled clinical trial.

**Table 3:** Overview of psychological measures and findings.

Main Author, Year	Measure	Intervention	*p* Value	Control	*p* Value	Comparison	*p* Value	Effect Size	Comments
**Stress, burnout, and recovery**									
**Stress**									
[31] Kjellgren, 2010	Stress and energy test	NA		NA		Difference	<0.01	NA	
[31] Kjellgren, 2010	Stress VAS	NA		NA		Difference	<0.05	NA	
[33] Largo-Wight, 2017	Perceived stress scale	NA		NA		Difference	<0.05	NA	
[38] Razani, 2018	Perceived stress scale	NA		NA		No difference		NA	
[42] Im, 2016	Stress responsive inventory	NA		NA		Difference	<0.05	NA	No baseline
[51] Marselle, 2014	Perceived stress scale	NA		NA		Difference	<0.001	0.22	
[53] Duvall, 2014	Perceived stress scale	NS						NA	
[54] Hofmann, 2017	Stress and coping inventory	NS						NA	
**Burnout**									
[61] Sahlin, 2014	Shirom-melamed burnout	Decrease	NA					NA	
***Recovery***									
[46] Tyrväinen, 2014	Recovery outcome scale	NA		NA		Difference	<0.01	0.53	
**Emotions**									
**Positive and negative affect**									
[27] Grazuleviciene, 2016	Positive and negative affect scale	NS		Decrease NA	<0.01			NA	
[35] Olafdottir, 2017	Positive and negative affect scale	NA		NA				NA	Results not reported
[36] Passmore, 2014	Positive and negative affect scale	NA		NA		Difference	<0.05	NA	
[37] Van den Berg, 2011	Positive and negative affect scale	Increase PA	<0.05	NS				NA	
[38] Fuegen, 2018	Positive and negative affect scale	NA		NA		Difference	<0.01	NA	
[46] Tyrväinen, 2014	Positive and negative affect scale	NA		NA		Difference	<0.01	Pa 0.43/Na 0.15	
[47] Berman, 2012	Positive and negative affect scale	improvement	<0.001	Improvement	<0.005	Difference	<0.001	NA	
[51] Marselle, 2014	Positive and negative affect scale	NA		NA		Difference	<0.001	Pa 0.24/Na 0.22	
[53] Duvall, 2014	Positive and negative affect scale	Improvement	<0.05–0.001					NA	
[55] Iwata, 2016	Positive and negative affect scale	NS		NA				NA	
[56] Marselle, 2016	Positive and negative affect scale	NA		NA		Difference	<0.05	NA	
**Mood**									
[32] Mao, 2011	Profile of mood states	NA		NA		Difference subscales	<0.05	NA	No baseline
[29] Niedermeyer, 2017	Mood survey scale	Improvement subscales	<0.001	NS				NA	
[34] Lee, 2010	Profile of mood states	Improvement	<0.01	improvement subscales	<0.01			NA	
[39] Gidlow, 2010	Profile of mood states	NA		NA		No difference		NA	
[41] Horiuchi, 2014	Profile of mood states	Improvement subscales	<0.05-0.01	improvement subscales	<0.01			NA	
[44] Song, 2015	Profile of mood states	NA		NA		Difference subscales	<0.05	NA	No baseline
[45] Stigsdotter, 2017	Profile of mood states	Improvement	<0.05	NS				NA	
[48] LI, 2016	Profile of mood states	Improvement subscales	<0.05-0.01	Recession subscales	<0.01			NA	
[54] Hofmann, 2017	Profile of mood states	NS		NS				NA	
[58] Ochiai, 2015a	Profile of mood states	Improvement subscales	<0.05					NA	
[59] Ochiai, 2015b	Profile of mood states	Improvement subscales	<0.01					NA	
**Anxiety**									
[29] Niedermeyer, 2017	State trait anxiety inventory	Decrease	<0.001	NS				NA	
[44] Song, 2015	State trait anxiety inventory	NA		NA		Difference	<0.01	NA	No baseline
[61] Sahlin, 2015	Beck anxiety inventory	Decrease	<0.005					NA	
[62] Yu, 2017	State trait anxiety inventory	Decrease	<0.01					NA	
**Depression**									
[50] Bang, 2017	Beck depression inventory	NA		NA		Difference	<0.001	NA	
[51] Marselle, 2014	Major depressive Inventory	NA		NA		Difference	<0.001	0.21	
[55] Iwata, 2016	Hamilton depression rating scale	NA		NA				NA	
[55] Iwata, 2016	Beck depression inventory	NA		NA				NA	
[61] Sahlin, 2015	Becks depression inventory	Decrease	<0.0001					NA	
**Combined measures**									
[52] Bird, 2015	Depression, anxiety, stress scale	Decrease	<0.001					NA	
Well-being, quality of life, and mental health									
[51] Marselle, 2014	Warwick Edinburgh mental well-being scale	NA		NA		Difference	<0.001	0.19	
[54] Hofmann, 2017	Mental health (SF12)	NS		NS				NA	
[56] Marselle, 2016	Single-item happiness scale	Increase	<0.001					NA	
[57] McCaffrey, 2016	Personal Growth Initiative Scale (PGIS)	Increase	<0.000					NA	
[57] McCaffrey, 2016	Quality of Life Scale	Increase	<0.001					NA	
[61] Sahlin, 2015	Psych. general well-being index	Increase	<0.0001					NA	

Abbreviations. NA: not available; NS: not significant; ES: effect size (Confidence intervals: NA). Stress VAS: stress visual analogue scale; Pa: positive affect; Na: negative affect. Note: If a measurement instrument included both positive and negative dimensions, the term ‘improved’ is used if the negative has decreased and the positive increased. Only validated measurement instruments are reported in the Table.

**Table 4:** Overview of physiological measures and effects.

Main Author, Year	Measure	Intervention	*p* Value	Control	*p* Value	Comparison	*p* Value	Comments
**Endocrine**								
**Cortisol**								
[27] Grazuleviciene, 2016	Salivary cortisol	NS		NS				
[28] Razani, 2018	Serum cortisol	NA		NA		Difference	<0.05	
[30] Han, 2016	Salivary cortisol	Decrease	<0.05	NS				
[32] Mao, 2011	Hair cortisol	NS		NS				
[34] Lee, 2010	Salivary cortisol	NS		NS				
[35] Olafdottir, 2017	Salivary cortisol	NA		NA		No difference		
[37] Van den Berg, 2011	Salivary cortisol	Decrease	<0.01	Decrease	<0.05			
[39] Gidlow, 2015	Salivary cortisol	Decrease	<0.01	Decrease	<0.01	No difference		
[43] Kobayashi, 2017	Salivary cortisol	NA		NA		Difference	<0.001	No baseline
[46] Tyrväinen, 2014	Salivary cortisol	Decrease	<0.01	Decrease	<0.01	No difference		
[49] Toda, 2013	Salivary cortisol	NS		NS				
[58] Ochiai, 2015a	Serum cortisol	Decrease	<0.01					
[59] Ochiai, 2015b	Salivary cortisol	Decrease	<0.05					
**Other**								
[48] Li, 2016	Stress hormones	Decrease	<0.01	Decrease	<0.01			
[41] Horiuchi, 2014	Salivia amylases	NA		NA		Difference	<0.05	
**Cardiovascular**								
**Heart rate variability**								
[34] Lee, 2010	HRV	Improved	*p* < 0.05	NA				
[35] Olafdottir, 2017	HRV	NA		NA				
[39] Gidlow, 2017	HRV	NS		NS				
[40] Gladwell, 2016	HRV	NA		NA		Difference	<0.05	
[41] Horiuchi, 2014	HRV	Decrease HR	<0.05	Decrease HR	<0.05	No Difference		
[44] Song, 2015	HRV	NA		NA		Difference	<0.01	
[45] Stigsdotter, 2017	HRV	Increase HF	<0.001	Increase HF	<0.001	No difference		
[50] Bang, 2017	HRV	NA		NA		Difference	<0.05	
[62] Yu, 2017	HRV	NS						
**Blood pressure**								
[27] Grazuleviciene, 2016	Blood pressure	Decrease DBP	<0.05	NS				
[31] Kjellgren, 2010	Blood pressure	NA		NA		No difference		
[34] Lee, 2010	Blood pressure	NS		NS				
[41] Huriuchi, 2014	Blood pressure	Decrease	*p* < 0.05	Decrease	*p* < 0.05	No difference		
[45] Stigsdotter, 2017	Blood pressure	Decrease	*p* < 0.05	Decease	*p* < 0.05	No difference		
[48] Li, 2016	Blood pressure	NS		NS				
[49] Toda, 2013	Blood pressure	Decrease	<0.05	NS				
[58] Ochiai, 2015a	Blood pressure	Decrease	<0.05					
[60] Ohe, 2017	Blood pressure	Decrease	<0.05					
[62] Yu, 2017	Blood pressure	Decrease	<0.01					
**Pulse rate**								
[31] Kjellgren, 2010	Pulse rate	NA		NA		No difference		
[34] Lee, 2010	Pulse rate	NA		NA		Difference	<0.01	
[48] Li, 2016	Pulse rate	NA		NA		Difference	<0.01	
[58] Ochiai, 2015a	Pulse rate	Decrease	<0.01					
[60] Ohe, 2017	Pulse rate	NS						
[62] Yu, 2017	Pulse rate	Decrease	<0.01					
**Immune system**								
[32] Mao, 2011	Serum PI	NA		NA		Difference	<0.05	
[32] Mao, 2011	Oxidative stress	NA		NA		Difference MDA	<0.001	
[42] Im, 2016	Blood serum	NA		NA		Difference	<0.05–0.001	No baseline

Abbreviations. NA: Not available; NS: not significant; HRV: heart rate variability; HR: heart rate; HF: high frequency; PI: pro-inflammatory; MDA: malondialdehyde. Note: If a measurement instrument involves both positive and negative dimensions, the word improved is used if the negative has decreased and the positive increased. Only validated measurement instruments are reported in the table. Only main results are reported. No effect sizes were reported; therefore this column is not entailed in the Table.
